# MLN4924 Promotes Self-Renewal of Limbal Stem Cells and Ocular Surface Restoration

**DOI:** 10.3390/jpm13030379

**Published:** 2023-02-21

**Authors:** Qingjian Li, Yankun Shen, Shinan Wu, Hong Wei, Jie Zou, Sanhua Xu, Qian Ling, Min Kang, Hui Huang, Xu Chen, Yi Shao

**Affiliations:** 1 Department of Ophthalmology, The First Affiliated Hospital of Nanchang University, Jiangxi Branch of National Clinical Research Center for Ocular Disease, Nanchang 330006, China; 2 Department of Ophthalmology, Xiang’an Hospital of Xiamen University, Fujian Provincial Key Laboratory of Ophthalmology and Visual Science, Eye Institute of Xiamen University, Xiamen University School of Medicine, Xiamen 361102, China; 3 Department of Ophthalmology, Huashan Hospital of Fudan University, Shanghai 200040, China; 4 Department of Ophthalmology and Visual Sciences, Maastricht University, 6200MA Maastricht, The Netherlands

**Keywords:** limbal stem cells, MLN4924, proliferation, differentiation, wound healing

## Abstract

Objective: To study the role of MLN4924 in corneal stem cell maintenance and corneal injury repair. Methods: In cell experiments, the Sprague–Dawley (SD) rat corneal epithelial cells were co-cultured with mitomycin C-inactivated mouse feeder cells in a supplemental hormonal epithelial medium (SHEM) with or without MLN4924. Cells were photographed using an optical microscope. Furthermore, we performed crystal violet, polymerase chain reaction (PCR), and immunofluorescence staining on limbal stem cells (LSCs). In animal experiments, we scraped the corneal epithelium with a central corneal diameter of 4 mm in SD rats. The area of the corneal epithelial defect was calculated by fluorescein sodium staining. Results: LSCs in the MLN4924 group had significantly proliferated. The MLN4924 treatment evidently enhanced the clone formation rate and clone area of LSCs. The expression levels of Ki67, p63, ABCG2, Bmi1, and C/EBPδ increased in LSCs after MLN4924 treatment, whereas the expression of K12 decreased. At 12 and 24 h after scraping, the corneal epithelium recovery rate in the eyes of the MLN4924-treated rats was accelerated. Conclusions: MLN4924 can maintain stemness, reduce differentiation, promote the proliferative capacity of rat LSCs, and accelerate corneal epithelial wound healing in SD rats.

## 1. Introduction

The cornea is the transparent portion of the eye, which focuses light on the retina for optical conduction [1]. The corneal epithelium is the outermost layer of the cornea and undergoes regular regeneration by limbal stem cells (LSCs). The epithelium maintains corneal transparency, protects the eyes from damage and infection, and helps the immune response by producing cytokines [2]. Thoft et al. proposed the X, Y, Z hypothesis of corneal epithelial reproduction. They assumed that during the corneal stable period, LSCs produced transient amplifying cells, which migrated inward and forward and became differential corneal epithelial cells [3]. Loss or dysfunction of LSCs leads to ingrowth of the conjunctival epithelium, corneal stromal neovascularization, and corneal opacity [4]. Ocular surface diseases that cause corneal stem cell defects, such as Stevens–Johnson syndrome, chemical burns, emission damage, widespread microbic infections, and hereditary conditions, may threaten vision and lead to blindness [5]. Data showed that corneal blindness ranked as the second leading inducement of global blindness, affecting 23 million people and thus increasing a substantial burden on medical resources [6]. The usual treatment is surgical transplantation of the donor cornea, which has been practiced for more than a century [7]. Moreover, LSC transplantation can also be used as an alternative therapy [8]. However, comprehensive studies have shown that LSCs have the crucial features of epithelial stem cells, which are infrequent, undifferentiated static cells with self-updating capacity, high proliferation potentiality, and tissue reproduction capacity [9]. LSCs respond to the conversion of corneal epithelial cells by differentiating into progenitor and proliferating cells, which split and relocate to the centric corneal basement layer to supplement the corneal epithelium [10]. This process promotes corneal wound healing by stimulating the proliferation of LSCs internally and externally and directing differentiation into corneal epithelial cells.

Ubiquitination adjusts steady-state protein stages mainly via the ubiquitin-proteasome targeted degradation system. Unlike ubiquitination, neddylation can regulate the functionality, structure, and orientation of its substrates [11]. Neddylation is the reversible covalent conjugation of the ubiquitin-like molecule NEDD8 (a developmental down-regulated protein 8 expressed by neuronal precursor cells) with the lysine residues of its substrates [12]. Analogous to ubiquitination, neddylation is initiated by a continuous cascade of NEDD8 activating enzyme E1, NEDD8 binding enzyme E2, and substrate-specific NEDD8-E3 ligase [13]. Neddylation is a highly conserved post-translational modification that allows cells to respond quickly and effectively to various stimuli [14]. Under physiological conditions, the level of neddylation is low. However, as a response to cellular stress or DNA damage, such as viral infection, inflammation, and cancer [15], activation of neddylation alters proteome homeostasis and affects stem cell proliferation [16]. MLN4924, also named Pevonedistat, is an especially tiny molecular suppressant of activating enzymes (NAE). It binds to the NAE active loci and forms a steady MLN4924–NEDD8 adduct, similar to adenylate–NEDD4, but suppresses further enzymatic processes, thereby obstructing the whole neddylation modification [17]. MLN4924 blocks the entire neddylation modification and inactivates all members of the Cullin-RING Ligase family by inhibiting E1. Cullin-RING ligase is often overexpressed in many types of human cancers [18]. MLN4924 exhibits impressive anti-cancer activity against a variety of human cancer cells in a wide range of preclinical environments by inducing growth arrest, apoptosis, autophagy, and senescence [19]. In addition, MLN4924 can promote wound healing induced by epidermal growth factor (EGF) in mouse skin. Low doses of MLN4924 can regulate the proliferation and differentiation of stem cells and have new applications in stem cell remedies [20].

This study investigated the effect of MLN4924 on the proliferation of LSCs and corneal wound healing. Our findings may provide a further understanding of the effects of MLN4924 on corneal repair management, therefore offering novel therapeutic approaches for treating corneal injuries.

## 2. Materials and Methods

### 2.1. Preparation of Feeder Cells

NIH 3T3 cells were obtained from the Cell Bank of the Chinese Academy of Sciences (Shanghai, China). The cells were maintained in Dulbecco’s modified Eagle’s medium (DMEM) supplemented with 10% fetal bovine serum (FBS) and 1% Penicillin-Streptomycin (PS). When NIH 3T3 cells reached 80–90% confluence, the culture medium was discarded, and the pre-prepared NIH 3T3 cell culture medium containing 10 μg/mL mitomycin was added. After 2 h of incubation at 37 °C, the medium was moved, the cells were washed with 1 × Hank’s Buffered Saline Solution (HBSS), replaced with a new NIH 3T3 cell culture medium, and placed back in the cell incubator with a CO_2_ concentration of 5% and a temperature of 37 °C. 

### 2.2. Isolation and Culture of LSCs

The limbus sample (~1 mm width) was obtained from the eyeballs of freshly sacrificed adult female Sprague–Dawley (SD) rats. Five rats were used for limbal tissue collection, and the cells from five rats were pooled together. The limbal tissue was washed 3 times with 1 × HBSS containing 10% FBS and 10% PS, 10 min each time. The clean limbal tissue was transferred to supplemental hormonal epithelial medium (SHEM) containing 2 U/mL Dispase II and then digested at 4 °C for 8 h. SHEM medium was made of DMEM/F12, 0.5% dimethyl sulfoxide (DMSO), 0.5 μg/mL hydrocortisone, 10 ng/mL mouse epidermal growth factor, 10 μg/mL insulin-transferrin-selenium-x, 5% FBS, and 1% PS. Subsequently, the isolated epithelial tissue was dissociated into single cells with 0.25% Trypsin-EDTA. The NIH 3T3 cells were also digested into single cells. Corneal epithelial cells were mixed with NIH 3T3 cells according to a density of 1000 cells/cm^2^ of limbal epithelial cells and 4.5 × 10^4^ cells/cm^2^ of NIH 3T3 cells and cultured with SHEM medium. To verify the effects of MLN4924 on LSCs, MLN4924 was added to the SHEM medium. Cells were photographed using an optical microscope (Leica, Wetzlar, Germany).

### 2.3. Crystal Violet Staining

After the LSCs had been cultured for 12 days, they were fixed with 4% paraformaldehyde solution at room temperature for 20 min and then washed 3 times with 1 × phosphate-buffered saline (PBS), 5 min each time. We added crystal violet staining solution at a concentration of 0.4%, stained at 25 °C for 30 min, and flushed 3 times with 1 × PBS buffer, 10 min each time. Finally, the sample was thoroughly dried in the air at room temperature and photographed with a camera (Canon, Tokyo, Japan).

### 2.4. Immunofluorescence Staining

After the LSCs had been cultured for 9 days, they were fixed with 4% paraformaldehyde solution at room temperature for 20 min and then washed 3 times with 1 × PBS, 5 min each time. The cells were incubated with cell permeabilization solution (0.2% TritonX-100) for 20 min at room temperature. After 1 h of blocking with 2% bovine serum albumin (BSA), the samples were incubated with the primary antibody (1:200 dilution) diluted in 1% BSA/PBS at 4 °C overnight. Rabbit anti-Ki67 (ab16667) and anti-K12 (ab185627) were purchased from Abcam (Cambridge, UK). After washing the primary antibody off, the cells were incubated with the secondary antibody (1:300 dilution) at room temperature for 1 h. Alexa Fluor 594-conjugated IgG (A21207) and Alexa Fluor 488-conjugated IgG (A21206) were purchased from Invitrogen (Eugene, OR, USA). The samples were finally mounted with H-1200 (containing DAPI) and photographed with a fluorescence microscope camera system (Leica, Wetzlar, Germany).

### 2.5. RNA Extraction and Polymerase Chain Reaction (PCR)

Total RNA was extracted from LSCs using Trizol reagent (ThermoFisher Scientific, Waltham, MA, USA). The RNA concentration was detected by a nucleic acid/protein analyzer (Beckman Coulter, Bria, CA, USA). Equal amounts of RNA were reverse-transcribed to cDNA by PrimeScript™ RT Master Mix (TaKaRa, Shiga, Japan) following the manufacturer’s protocol. The PCR was performed using Taq Pro Universal SYBR qPCR Master Mix (Vazyme, Nanjing, Jiangsu, Chnia) on a LightCycler^®^ 96 instrument (Roche, Basel, Switzerland). The PCR program used was as follows: preincubation at 95 °C for 30 s, followed by 40 cycles of denaturation at 95 °C for 10 s, annealing at 60 °C for 20 s, extension at 72 °C for 20 s, and final melting at 95 °C for 10 s, 65 °C for 60 s, and 97 °C for 1 s. Results were calculated using the 2^-△△CT^ method to evaluate relative gene expression levels. The primers were: β-actin, 5’-CACCCGCGAGTACAACCTTC-3’ (forward) and 5’-CCCATACCCACCATCACACC-3’ (reverse). Ki67, 5’-CTCAGCTCCTGCCTGTTTGG-3’ (forward) and 5’-ACTTGAGTTGGATTGGCGGA-3’ (reverse); p63, 5’-CAGCACACGATCGAGACGTA-3’ (forward), and 5’-CGGGACTCCACAAGCTCATT-3’ (reverse); ABCG2, 5’-GTAGGTCGGTGTGCGAGTCAG-3’ (forward), and 5’-AAGGCCGTTCTTGTTTCTCTGT-3’ (reverse); Bmi1, 5’-AGCGGTGGCTGGATGC-3’ (forward), and 5’-GGTCTCCAAGTAACGCACAA-3’ (reverse); K12, 5’-AGCTAACGCGGAACTGGAAA-3’ (forward), and 5’-CTCTCCGCTCTTGGTGAGGT-3’ (reverse).

### 2.6. Western Blotting

Proteins from LSCs were extracted with a cold lysis buffer composed of protease and phosphatase inhibitors (ThermoFisher Scientific, Waltham, MA, USA). Protein concentration was measured by a BAC protein assay kit (ThermoFisher Scientific, Waltham, MA, USA). Equal amounts of protein extracts (15 μg) were subjected to electrophoresis on 10% acrylamide gels and then transferred to polyvinylidene difluoride membranes (Roche, Basel, Switzerland). After being blocked in 5% BSA for 1 h at 25 °C, the membranes were incubated overnight at 4 °C with primary antibodies for anti-p63 (1:1000 dilution, ab124762, Abcam, Cambridge, UK), C/EBPδ (1:1000 dilution, ab65081, Abcam, Cambridge, UK), and K12 (ab185627, Abcam, Cambridge, UK). After 3 washes with Tris-buffered saline containing 0.05% Tween-20 for 10 min each, the membranes were incubated with HRP-conjugated goat anti-rabbit IgG secondary antibody (1: 5000 dilution, 31460, ThermoFisher Scientific, Waltham, MA, USA) for 1 h at room temperature. An HRP-conjugated mouse anti-β-actin antibody (1:1000 dilution, A3854, Sigma-Aldrich, Saint Louis, MO, USA) was used for protein quantification. The results were detected by an enhanced chemiluminescence reagent kit (NCM Biotech, Suzhou, China) and recorded by the transilluminator (Bio-Rad, Hercules, CA, USA).

### 2.7. Corneal Epithelial Scratch Model

Animal experiments were authorized by the Institutional Animal Care and Use Committee of Xiamen University School of Medicine. All animals were treated in accordance with national and international animal welfare rules. Under the surgical microscope, a corneal trephine with a diameter of 4 mm was used to align the corneas from 180 to 200 g female SD rats. The corneal trephine was rotated to make a circular incision in the corneal epithelium. A corneal scraper was used to remove the epithelium in the corneal ring incision. To verify the effect of MLN4924 on the healing rate of the corneal epithelium, eye drops (10 μL) containing 1 μM MLN4924 or 0.1% DMSO were topically administered 4 times daily 24 h before the wound healing assay for a consecutive 2-day period. Corneal fluorescein sodium staining and a slit lamp microscope (66 Vision-Tech, Suzhou, Jiangsu, China) were used to calculate the area of the corneal epithelial defect.

### 2.8. Statistical Analysis

All statistical analyses were performed with the GraphPad Prism software (San Diego, CA, USA). Data are presented as mean ± standard deviation (SD). A one-way analysis of variance, or *t*-test, was used to assess the distinctions between the groups. *p* < 0.05 indicated statistically significant differences.

## 3. Results

### 3.1. MLN4924 Promotes Clone Formation Rate and Clone Area of LSCs 

**Figure 1:**
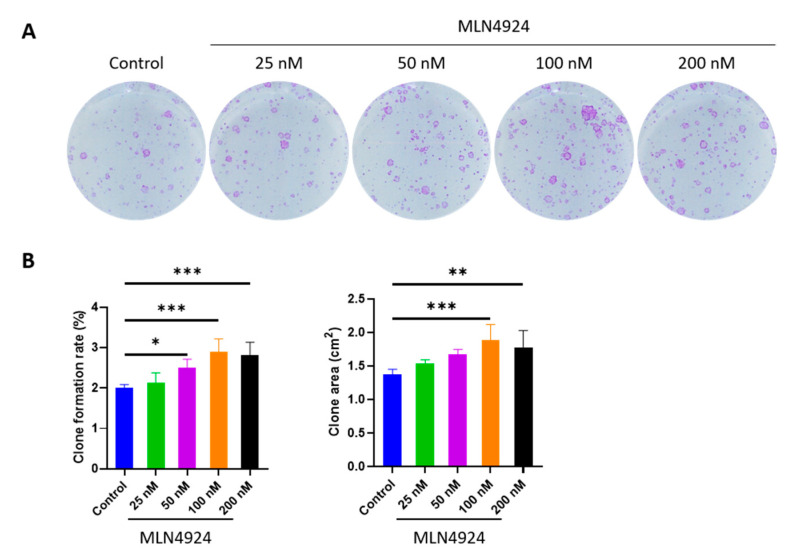
(**A**) The crystal violet staining showed that the clone formation rate and clone area of LSCs increased after MLN4924 treatment. (**B**) The statistical analysis showed significant differences among the groups concerning the clone formation rate and clone area of LSCs. Out of the different concentrations of MLN4924, 100 nM showed the best results. All of the experiments were independently repeated five times. * *p* < 0.05, ** *p* < 0.01, and *** *p* < 0.001.

The clones were visualized by crystal violet staining. The clone formation rate was calculated as follows: clone formation rate (%) = number of clones/number of seeded cells × 100%. The results showed that the clone formation rate and clone area of LSCs increased after MLN4924 treatment, as shown in Figure 1. Out of the different concentrations of MLN4924, 100 nM showed the best results.

### 3.2. MLN4924 Improves the Proliferative Capacity of LSCs In Vitro

**Figure 2:**
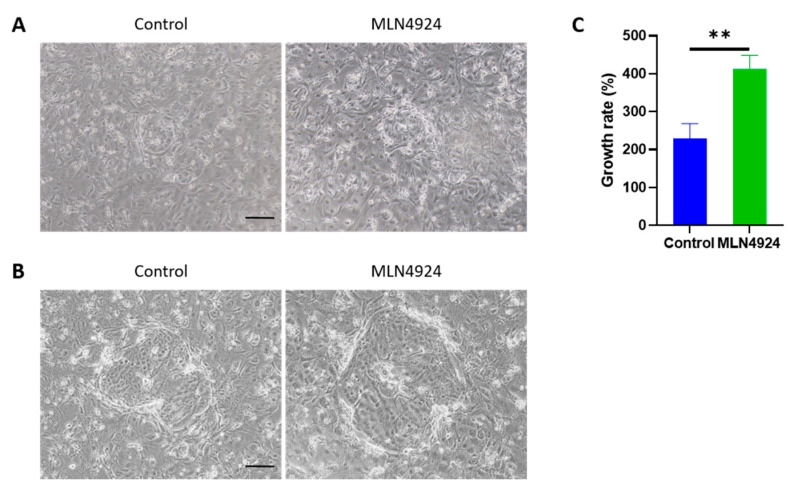
MLN4924 improved the proliferative potential of LSCs in vitro. (**A**) Before treatment. (**B**) Two days after treatment. Scale bar: 100 μm. (**C**) The statistical analysis showed a significant difference between the control group and the MLN4924 group concerning the growth rate of LSCs. All the experiments were independently repeated three times. ** *p* < 0.01.

To examine the effect of MLN4924 on LSCs, 100 nM MLN4924 was used for the in vitro experiments. The growth rate of LSCs in a clone was calculated according to the following formulas: growth rate (%) = (number of LSCs 2 days after treatment − number of LSCs before treatment)/number of LSCs before treatment × 100%. It could be observed under an optical microscope that after 2 days of treatment, the LSCs of the MLN4924 group significantly proliferated when compared with the control group (Figure 2).

### 3.3. MLN4924 Increases the Expression Levels of Ki67 and p63 in LSCs

**Figure 3:**
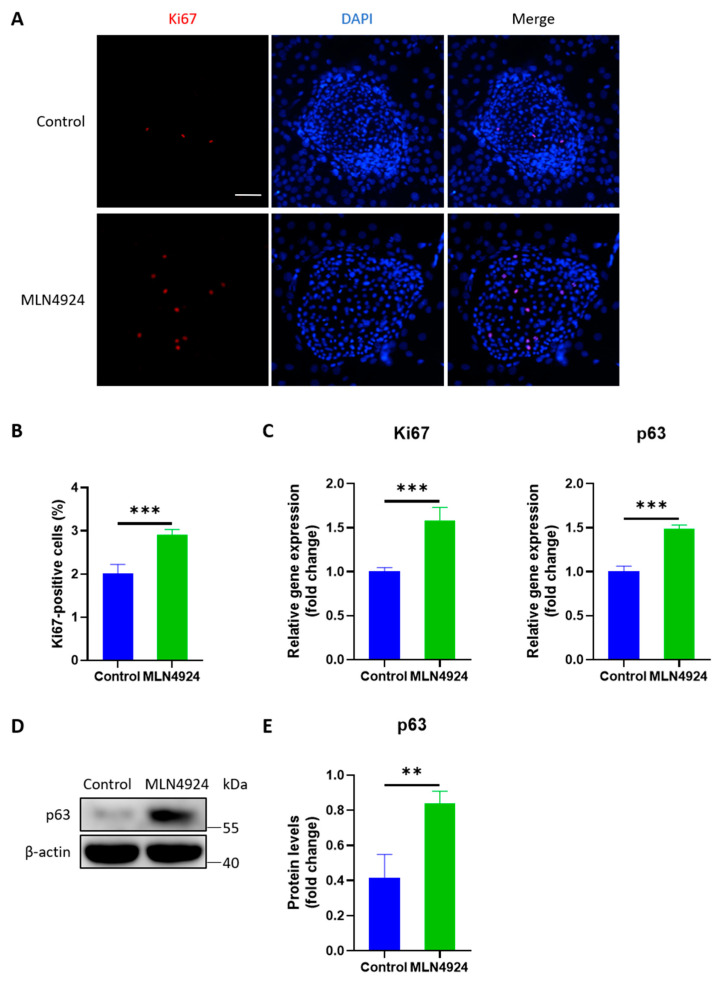
(**A**) Immunofluorescence staining showed that Ki67 increased in LSCs after MLN4924 treatment. (**B**) Statistical analysis revealed a higher percentage of Ki67-positive cells in the MLN4924 group compared to the control group. (**C**) The mRNA expression levels of Ki67 and p63 in LSCs increased after MLN4924 treatment. (**D**) Western blotting showed that the p63 protein level in the MLN4924 group was higher than in the control group. (**E**) MLN4924 treatment significantly increased the protein level of p63 in LSCs. All of the experiments were independently repeated four times. ** *p* < 0.01 and *** *p* < 0.001. Scale bar: 100 μm.

The Ki67 index was calculated as the percentage of Ki67-positive cells relative to the total number of LSCs within the same clone. Immunofluorescence staining showed that Ki67 increased in LSCs after MLN4924 treatment. PCR analysis showed significant increases in Ki67 and p63 expression in the MLN4924 group. Western blotting showed that the p63 protein level in the MLN4924 group was higher than that in the control group, as shown in Figure 3. 

### 3.4. MLN4924 Enhances the Preservation of Stemness of LSCs

**Figure 4:**
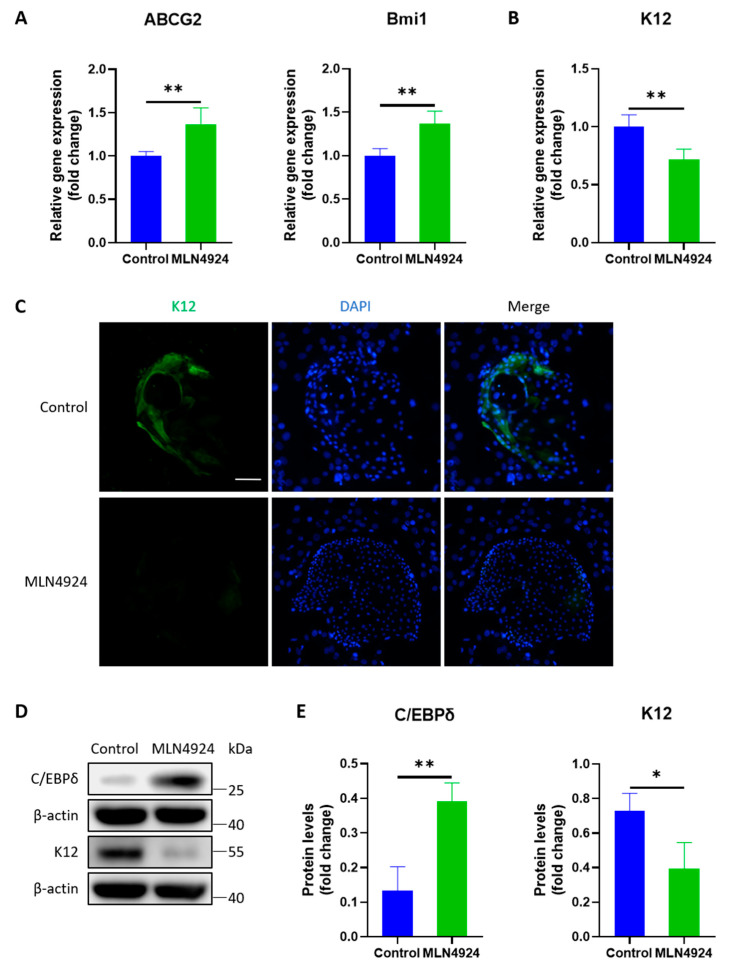
(**A**) Compared with the control group, the MLN4924 group had increased expression levels of ABCG2 and Bmi1. (**B**) The K12 mRNA levels decreased after MLN4924 treatment. (**C**) Immunofluorescence staining showed that MLN4924 suppressed the expression of K12. (**D**) Western blotting showed that the C/EBPδ protein expression level increased in LSCs after MLN4924 treatment, whereas the protein level of K12 decreased. (**E**) Statistical analysis revealed a higher protein expression level of C/EBPδ and a lower protein level of K12. All of the experiments were independently repeated four times. * *p* < 0.05 and ** *p* < 0.01. Scale bar: 100 μm.

PCR analysis revealed a higher expression level of C/EBPδ mRNA and a lower level of K12 mRNA in the MLN4924 group. Furthermore, immunofluorescence staining showed that K12 decreased in the MLN4924 group. Western blotting showed that the C/EBPδ protein expression level increased in LSCs after MLN4924 treatment, whereas the protein level of K12 decreased. The results reflect that MLN4924 can maintain stemness and reduce the differentiation of LSCs, as shown in Figure 4.

### 3.5. MLN4924 Promotes Corneal Wound Healing

**Figure 5:**
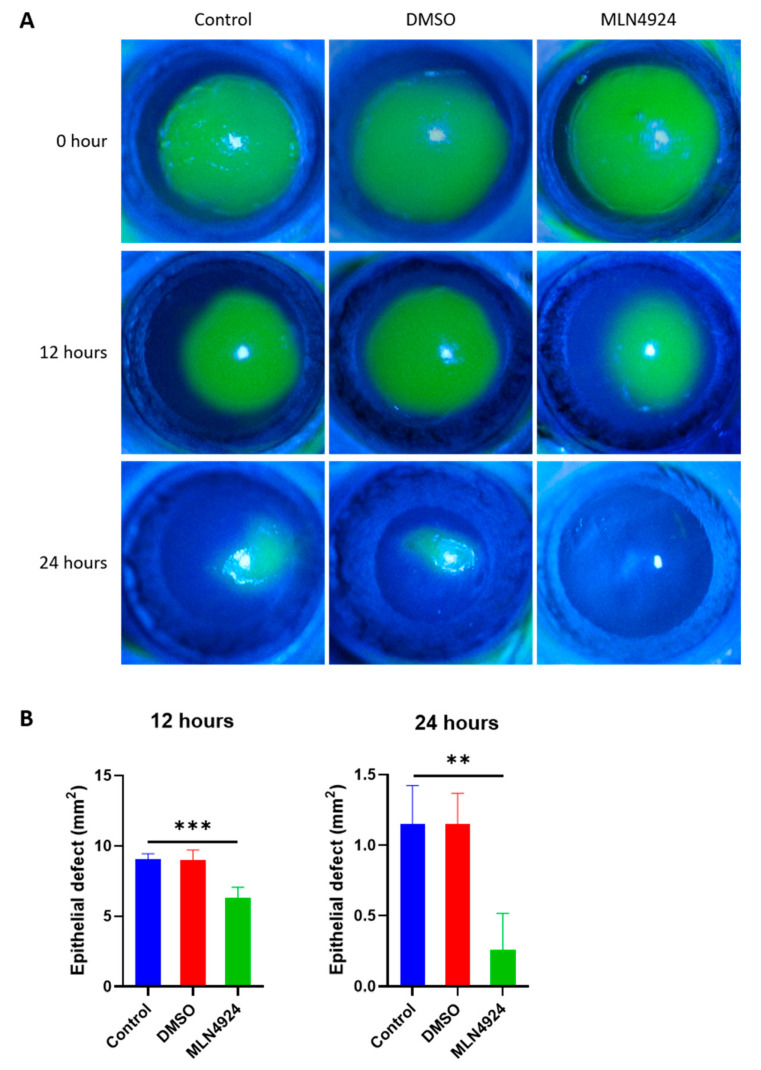
(**A**) The recovery rate of corneal epithelium in MLN4924 eyes was accelerated at 12 and 24 h after scraping the epithelium with a central corneal diameter of 4 mm in SD rats. (**B**) Statistical analysis showed that the recovery rate of corneal epithelium in MLN4924 eyes was significantly different from that in the control and DMSO groups. All of the experiments were independently repeated three times. ** *p* < 0.01 and *** *p* < 0.001.

The corneal epithelium is constantly renewed by LSCs that exclusively reside in the corneoscleral junction. At 12 and 24 h after scraping the epithelium with a diameter of 4 mm in the central cornea of SD rats, the recovery rate of corneal epithelium in the MLN4924 eye was accelerated, as shown in Figure 5.

## 4. Discussion

Limbus is the interim zone between the pellucid cornea and opaque sclera [21]. LSCs are mature stem cells that further differentiate into the corneal epithelium. Functional LSCs are crucial for maintaining the entire corneal surface and corneal transparency. The normal limbus and LSCs act as a barrier to prevent conjunctival epithelial cells from invading the cornea [22]. LSCs are quiescent stem cells that can be activated to divide symmetrically and asymmetrically, producing proliferative progenitor cells that produce mature corneal epithelial cells [23]. Our results demonstrated that the addition of exogenous MLN4924 in vivo and in vitro could effectively maintain the stemness of LSCs and promote LSC proliferation. This finding provides a new idea for constructing a limbal equivalent for ocular surface reconstruction.

The limbus acts as a barrier between the cornea and the conjunctiva, blocking conjunctivalization and neovascularization of the cornea [24]. The asymmetric division of an LSC can produce a limbal stem daughter cell and a transient amplifying cell. During migration, LSCs differentiate until they become squamous cells and detach from the corneal surface [25]. Many studies have proven the barrier function of the corneal limbus. Chung et al. researched the orientation of corneal epithelial stem cells in rats during their growth and proposed that stem cells were distributed in the basal layer of the cornea and limbal epithelium. When the cornea matures, the epithelial stem cells become concentrated in the limbus [26]. By continuously culturing cells from the ocular surface, Pellegrini et al. discovered that cells from the limbal biopsy experienced 85 doublings, while cells from the central cornea failed to survive in continuous culture, the result of which proved that cells in the marginal zone of the corneal epithelium possessed strong proliferative capability [27]. Stem cells at the edge of the Vogt fence are particularly abundant. These fences are radial fibrovascular ridges, primarily centered on the upper and lower edges [28]. LSCs and the limbal microenvironment play an important role in maintaining corneal stability and corneal immunity. The depletion of LSCs and the devastation of stem cell conditions may alter corneal stability [24]. Under pathological circumstances, the conjunctival epithelium can shift at the edge of the limbus; in the worst case, it can lead to inflammatory fibrovascular conjunctival centripetal pacing [29]. In recent decades, scientists have promoted more efficient methods for corneal injury, including continuous treatment, surgical technology, and advanced transplantation methods [30]. Our study proposes that specific ubiquitin-activating enzyme inhibitors could promote the proliferation of LSCs, which provides a new way of treating corneal blindness. 

Recent studies have demonstrated that neddylation is associated with many pathophysiological states, such as adipogenesis, cardiac homeostasis, synapse formation, and tumor development [11,31,32,33]. MLN4924 is a specific inhibitor of NAE1. NAE1 can lead to an increase in the autophagy flux of vascular smooth muscle cells [34,35]. MLN4924 has shown good prospects for the treatment of a variety of malignant tumors [36]. It has also exhibited anti-tumor effects in preclinical xenograft models of solid tumors and blood cancers, including acute myeloid leukemia and lymphoma models [37]. Neddylation regulates the tumor microenvironment composed of tumor cells, immune cells, etc. and plays a role in tumor progression [38]. NEDD acylation can inhibit the anti-tumor activity of tumor-associated macrophages, T cells, and dendritic cells [39]. MLN4924 is undergoing several clinical studies to evaluate its anticancer effects on solid tumors and leukemia. Five phase I clinical trials have been completed, suggesting that MLN4924 alone or in combination with chemotherapy is safe and shows effective therapeutic effects [40]. Moreover, the potential anticancer mechanism of MLN4924 is its inhibitory effect on NAE activity by binding to NAE to produce a covalent NEDD8-MLN4924-adduct [41]. Therefore, MLN4924 can effectively prevent the neddylation of all Cullins, leading to the accumulation of its substrates, which in turn triggers DNA replication stress, DNA damage response, cell cycle arrest, apoptosis, autophagy, and senescence [42,43]. Neddylation pathway components and CRL1/SCF E3 ligase are potential anticancer biomarkers, and MLN4924 can be a potential drug for cancer treatment [44]. The data showed that MLN4924 significantly inhibited the growth of renal cancer cells by blocking Cullin1 neddylation and subsequent substrate accumulation. In addition, MLN4924 prevented the migration of renal cancer cells by up-regulating E-cadherin and inhibiting Vimentin [45]. Recent studies have also used MLN4924 to block NAE1 and inhibit neddylation, which can eventually block terminal myoblast differentiation [46]. Studies have shown that sRANKL-activated TRAF6 leads to the automatic amplification of NFAT-c1 and promotes osteoclast differentiation [47]. MLN4924 can inhibit the levels of TRAF6, phosphorylated ERK, phosphorylated p38MAPK, and phosphorylated JNK. This means that in the early stages, neddylation inhibition can reduce the downstream signal of the sRANKL pathway. In addition, μCT analysis of trabecular bone microstructure showed deterioration of bone microstructure in the OVX/sRANKL group, which was reversed by MLN4924, indicating that it can inhibit sRANKL-induced bone loss and may be a new target for the treatment of osteoporosis [48]. For self-renewing hematopoietic stem cells, MLN4924 regulates ubiquitination and neddylation and promotes hematopoietic stem cell proliferation through cyclin-dependent kinase inhibitors [49]. Therefore, we believe that MLN4924 can promote corneal healing by increasing the proliferation of LSC while reducing their differentiation. Previous studies have shown that the mitotic marker Ki67 of LSCs determined by immunohistochemical staining is an important proliferation marker, and K12 is an important differentiation marker [50,51,52]. C-MYC is one of four Yamanaka factors that reprogram fibroblasts into induced pluripotent stem cells [53]. It portrays an essential character in the self-renewal of stem cells [54]. The researchers observed a dose-dependent increase in c-MYC after MLN4924 treatment in multiple cell lines and determined that there was a clear causal relationship between c-MYC accumulation and the stimulating effect of MLN4924. They found that MLN4924 treatment caused c-MYC accumulation in wild-type cells, tested the response of isogenic lines to MLN4924 and found that MLN49244 could still stimulate tumor sphere formation in FBXW7 empty cells [20]. Therefore, the stimulation effect of MLN4924 may be mediated by the c-MYC/FBXW7 axis. Ki67 is usually used as a prognostic marker in clinical practice due to its specificity to proliferating cells and its ability to detect cell cycles. The positive staining of Ki67 protein and other markers in the tumor samples of patients can be used to grade the primary tumor and metastasis [55,56]. Moreover, Ki67 staining also has prognostic value in predicting cancer survival and recurrence [57]. The p63 gene is a member of the p53 family and is essential for the development of epithelial cells and the regulation of epithelial cell proliferation and differentiation. The p63 gene exists in two different subtypes, TAp63 and ΔNp63 [58]. Among them, ΔNp63 portrays an essential character in HNSCC cell survival and inhibits the p73-dependent pro-apoptotic transcription program [59]. The ΔNp63α/HDAC1/2 complex is also considered to be an important tumor maintenance factor [60]. ABCG2 is a well-characterized ABC transporter. Some cell experiments have confirmed that the ABCG2 level is a key determinant of cell sensitivity to MLN4924 [61]. It has been reported that MLN4924 may be a potential substrate of ABCG2, which can stimulate the ATPase activity of ABCG2 in a concentration-dependent manner [62]. The data from cell experiments showed that the sensitivity of ABCG2-overexpressing cells to MLN4924 was significantly reduced. Knockdown of ABCG2 could partially restore the sensitivity of MLN4924, indicating that it played an important role in controlling the sensitivity of MLN4924 [63]. Our experiments showed that the ABCG2 of LSCs increased after MLN4924 treatment, which may subsequently cause stem cell resistance to MLN4924, which is also a situation that needs our attention in clinical application. Bmi1 is the core component of the polycomb repressor complex, which mediates gene silencing through the monoubiquitination of histone H2A [64]. In addition, Bmi1 is an important stem cell self-renewal factor [65]. More importantly, researchers found that circ_001680 could promote the number of cancer stem cells in colorectal cancer by regulating Bmi1 through miR-340 [66]. Our cytological experiments showed that Ki67, p63, ABCG2, and Bmi1 of SD rat LSCs increased and K12 decreased after MLN4924 treatment, indicating that MLN4924 could maintain the stemness of LSCs and reduce differentiation. Based on the results from animal experiments, we observed that MLN4924 could accelerate corneal epithelial recovery. This finding indicated that MLN4924 could promote the migration of limbal epithelial cells and wound healing. This not only provides a broader clinical prospect for MLN4924 but also offers a more feasible treatment for corneal limbus injury.

## 5. Conclusions

In this study, through extracorporeal cell experiments and intracorporeal animal experiments, we confirmed that MLN4924 could improve the proliferative capacity of rat limbal epithelial stem cells and promote corneal wound healing in SD rats. Therefore, MLN4924 may become an underlying therapeutic objective for ocular surface renovation and reestablishment in clinical applications.

## Data Availability

Data are contained within the article.

## References

[1] Osei-Bempong C., Figueiredo F.C., Lako M. (2013). The limbal epithelium of the eye—A review of limbal stem cell biology, disease and treatment. Bioessays.

[2] DelMonte D.W., Kim T. (2011). Anatomy and physiology of the cornea. J. Cataract. Refract. Surg..

[3] Thoft R.A., Friend J. (1983). The X, Y, Z hypothesis of corneal epithelial maintenance. Investig. Ophthalmol Vis Sci..

[4] Sasamoto Y., Ksander B.R., Frank M.H., Frank N.Y. (2018). Repairing the corneal epithelium using limbal stem cells or alternative cell-based therapies. Expert. Opin. Biol. Ther..

[5] Vazirani J., Nair D., Shanbhag S., Wurity S., Ranjan A., Sangwan V. (2018). Limbal Stem Cell Deficiency-Demography and Underlying Causes. Am. J. Ophthalmol..

[6] Oliva M.S., Schottman T., Gulati M. (2012). Turning the tide of corneal blindness. Indian J. Ophthalmol..

[7] Chirila T.V., Harkin D. (2009). Biomaterials and Regenerative Medicine in Ophthalmology.

[8] Kolli S., Ahmad S., Mudhar H.S., Meeny A., Lako M., Figueiredo F.C. (2014). Successful application of ex vivo expanded human autologous oral mucosal epithelium for the treatment of total bilateral limbal stem cell deficiency. Stem Cells.

[9] Gonzalez G., Sasamoto Y., Ksander B.R., Frank M.H., Frank N.Y. (2018). Limbal stem cells: Identity, developmental origin, and therapeutic potential. Wiley Interdiscip. Rev. Dev. Biol..

[10] Zhou M., Li X.M., Lavker R.M. (2006). Transcriptional profiling of enriched populations of stem cells versus transient amplifying cells. A comparison of limbal and corneal epithelial basal cells. J. Biol. Chem..

[11] Zhou L., Zhang W., Sun Y., Jia L. (2018). Protein neddylation and its alterations in human cancers for targeted therapy. Cell Signal..

[12] Xirodimas D.P. (2008). Novel substrates and functions for the ubiquitin-like molecule NEDD8. Biochem. Soc. Trans..

[13] Zhao Y., Morgan M.A., Sun Y. (2014). Targeting Neddylation pathways to inactivate cullin-RING ligases for anticancer therapy. Antioxid. Redox. Signal..

[14] Liddy K.A., White M.Y., Cordwell S.J. (2013). Functional decorations: Post-translational modifications and heart disease delineated by targeted proteomics. Genome Med..

[15] Bailly A.P., Perrin A., Serrano-Macia M., Maghames C., Leidecker O., Trauchessec H., Martinez-Chantar M.L., Gartner A., Xirodimas D.P. (2019). The Balance between Mono- and NEDD8-Chains Controlled by NEDP1 upon DNA Damage Is a Regulatory Module of the HSP70 ATPase Activity. Cell Rep..

[16] Zou T., Zhang J. (2021). Diverse and pivotal roles of neddylation in metabolism and immunity. FEBS J..

[17] Soucy T.A., Smith P.G., Milhollen M.A., Berger A.J., Gavin J.M., Adhikari S., Brownell J.E., Burke K.E., Cardin D.P., Critchley S. (2009). An inhibitor of NEDD8-activating enzyme as a new approach to treat cancer. Nature.

[18] Zhao Y., Sun Y. (2013). Cullin-RING Ligases as attractive anti-cancer targets. Curr. Pharm. Des..

[19] Xie M., Guo H., Lou G., Yao J., Liu Y., Sun Y., Yang Z., Zheng M. (2021). Neddylation inhibitor MLN4924 has anti-HBV activity via modulating the ERK-HNF1α-C/EBPα-HNF4α axis. J. Cell Mol. Med..

[20] Zhou X., Tan M., Nyati M.K., Zhao Y., Wang G., Sun Y. (2016). Blockage of neddylation modification stimulates tumor sphere formation in vitro and stem cell differentiation and wound healing in vivo. Proc. Natl. Acad. Sci. USA.

[21] Skeens H.M., Brooks B.P., Holland E.J. (2011). Congenital aniridia variant: Minimally abnormal irides with severe limbal stem cell deficiency. Ophthalmology.

[22] Ramos T., Scott D., Ahmad S. (2015). An Update on Ocular Surface Epithelial Stem Cells: Cornea and Conjunctiva. Stem Cells Int..

[23] Robertson S.Y.T., Roberts J.S., Deng S.X. (2021). Regulation of Limbal Epithelial Stem Cells: Importance of the Niche. Int. J. Mol. Sci..

[24] Notara M., Lentzsch A., Coroneo M., Cursiefen C. (2018). The Role of Limbal Epithelial Stem Cells in Regulating Corneal (Lymph)angiogenic Privilege and the Micromilieu of the Limbal Niche following UV Exposure. Stem Cells Int..

[25] Yoon J.J., Ismail S., Sherwin T. (2014). Limbal stem cells: Central concepts of corneal epithelial homeostasis. World J. Stem Cells.

[26] Chung E.H., Bukusoglu G., Zieske J.D. (1992). Localization of corneal epithelial stem cells in the developing rat. Investig. Ophthalmol. Vis. Sci..

[27] Pellegrini G., Golisano O., Paterna P., Lambiase A., Bonini S., Rama P., De Luca M. (1999). Location and clonal analysis of stem cells and their differentiated progeny in the human ocular surface. J. Cell Biol..

[28] Goldberg M.F., Bron A.J. (1982). Limbal palisades of Vogt. Trans. Am. Ophthalmol. Soc..

[29] Di Girolamo N. (2015). Moving epithelia: Tracking the fate of mammalian limbal epithelial stem cells. Prog. Retin. Eye Res..

[30] Gericke A., Wasielica-Poslednik J., Zimmermann M., Musayeva A. (2019). Expansion und Transplantation limbaler Stammzellen zur Regeneration der kornealen Oberfläche [Expansion and Transplantation of Limbal Stem Cells for Corneal Surface Regeneration]. Klin. Monbl. Augenheilkd..

[31] Park H.S., Ju U.I., Park J.W., Song J.Y., Shin D.H., Lee K.H., Jeong L.S., Yu J., Lee H.W., Cho J.Y. (2016). PPARγ neddylation essential for adipogenesis is a potential target for treating obesity. Cell Death Differ..

[32] Zou J., Ma W., Li J., Littlejohn R., Zhou H., Kim I.M., Fulton D.J.R., Chen W., Weintraub N.L., Zhou J. (2018). Neddylation mediates ventricular chamber maturation through repression of Hippo signaling. Proc. Natl. Acad. Sci. USA.

[33] Li L., Cao Y., Wu H., Ye X., Zhu Z., Xing G., Shen C., Barik A., Zhang B., Xie X. (2016). Enzymatic Activity of the Scaffold Protein Rapsyn for Synapse Formation. Neuron.

[34] Deng Q., Zhang J., Gao Y., She X., Wang Y., Wang Y., Ge X. (2017). MLN4924 protects against bleomycin-induced pulmonary fibrosis by inhibiting the early inflammatory process. Am. J. Transl. Res..

[35] Ai T.J., Sun J.Y., Du L.J., Shi C., Li C., Sun X.N., Liu Y., Li L., Xia Z., Jia L. (2018). Inhibition of neddylation by MLN4924 improves neointimal hyperplasia and promotes apoptosis of vascular smooth muscle cells through p53 and p62. Cell Death Differ..

[36] Bhatia S., Pavlick A.C., Boasberg P., Thompson J.A., Mulligan G., Pickard M.D., Faessel H., Dezube B.J., Hamid O. (2016). A phase I study of the investigational NEDD8-activating enzyme inhibitor pevonedistat (TAK-924/MLN4924) in patients with metastatic melanoma. Investig. New Drugs.

[37] Khalife J., Radomska H.S., Santhanam R., Huang X., Neviani P., Saultz J., Wang H., Wu Y.-Z., Alachkar H., Anghelina M. (2015). Pharmacological targeting of miR-155 via the NEDD8-activating enzyme inhibitor MLN4924 (Pevonedistat) in FLT3-ITD acute myeloid leukemia. Leukemia.

[38] Whiteside T.L. (2008). The tumor microenvironment and its role in promoting tumor growth. Oncogene.

[39] Zhou L., Jiang Y., Luo Q., Li L., Jia L. (2019). Neddylation: A novel modulator of the tumor microenvironment. Mol. Cancer.

[40] Pellegrino N.E., Guven A., Gray K., Shah P., Kasture G., Nastke M.D., Thakurta A., Gesta S., Vishnudas V.K., Narain N.R. (2022). The Next Frontier: Translational Development of Ubiquitination, SUMOylation, and NEDDylation in Cancer. Int. J. Mol. Sci..

[41] Petroski M.D. (2010). Mechanism-based neddylation inhibitor. Chem. Biol..

[42] Liao H., Liu X.J., Blank J.L., Bouck D.C., Bernard H., Garcia K., Lightcap E.S. (2011). Quantitative proteomic analysis of cellular protein modulation upon inhibition of the NEDD8-activating enzyme by MLN4924. Mol. Cell Proteom..

[43] Milhollen M.A., Narayanan U., Soucy T.A., Veiby P.O., Smith P.G., Amidon B. (2011). Inhibition of NEDD8-activating enzyme induces rereplication and apoptosis in human tumor cells consistent with deregulating CDT1 turnover. Cancer Res..

[44] Zhang Y., Shi C.C., Zhang H.P., Li G.Q., Li S.S. (2016). MLN4924 suppresses neddylation and induces cell cycle arrest, senescence, and apoptosis in human osteosarcoma. Oncotarget.

[45] Tong S., Si Y., Yu H., Zhang L., Xie P., Jiang W. (2017). MLN4924 (Pevonedistat), a protein neddylation inhibitor, suppresses proliferation and migration of human clear cell renal cell carcinoma. Sci. Rep..

[46] Zhou H., Su H., Chen W. (2021). Neddylation Regulates Class IIa and III Histone Deacetylases to Mediate Myoblast Differentiation. Int. J. Mol. Sci..

[47] Li L., Liu B., Dong T., Lee H.W., Yu J., Zheng Y., Gao H., Zhang Y., Chu Y., Liu G. (2013). Neddylation pathway regulates the proliferation and survival of macrophages. Biochem. Biophys. Res. Commun..

[48] Wu M.H., Hsu W.B., Chen M.H., Shi C.S. (2022). Inhibition of Neddylation Suppresses Osteoclast Differentiation and Function In Vitro and Alleviates Osteoporosis In Vivo. Biomedicines.

[49] Albayrak E., Uslu M., Akgol S., Tuysuz E.C., Kocabas F. (2021). Small molecule-mediated modulation of ubiquitination and neddylation improves HSC function ex vivo. J. Cell Physiol..

[50] Kammergruber E., Rahn C., Nell B., Gabner S., Egerbacher M. (2019). Morphological and immunohistochemical characteristics of the equine corneal epithelium. Vet. Ophthalmol..

[51] Liu T., Wang Y., Duan H.Y., Qu M.L., Yang L.L., Xu Y.Y., Zang X.J., Zhou Q.J. (2012). Effects of preservation time on proliferative potential of human limbal stem/progenitor cells. Int. J. Ophthalmol..

[52] Busin M., Breda C., Bertolin M., Bovone C., Ponzin D., Ferrari S., Barbaro V., Elbadawy H.M. (2016). Corneal Epithelial Stem Cells Repopulate the Donor Area within 1 Year from Limbus Removal for Limbal Autograft. Ophthalmology.

[53] Takahashi K., Yamanaka S. (2006). Induction of pluripotent stem cells from mouse embryonic and adult fibroblast cultures by defined factors. Cell.

[54] Quinn L.M., Secombe J., Hime G.R. (2013). Myc in stem cell behaviour: Insights from Drosophila. Adv. Exp. Med. Biol..

[55] Aporowicz M., Czopnik P., Kubicka E., Piotrowska A., Dziegiel P., Bolanowski M., Domoslawski P. (2019). Minichromosome Maintenance Proteins MCM-3, MCM-5, MCM-7, and Ki-67 as Proliferative Markers in Adrenocortical Tumors. Anticancer Res..

[56] da Silva J.N.L., Ranzi A.D., Carvalho C.T., Scheide T.V., Strey Y.T.M., Graziottin T.M., Bica C.G. (2020). Cell Cycle Markers in the Evaluation of Bladder Cancer. Pathol. Oncol. Res..

[57] Pouget C., Hergalant S., Lardenois E., Lacomme S., Houlgatte R., Carpentier C., Dehais C., Rech F., Taillandier L., Sanson M. (2020). Ki-67 and MCM6 labeling indices are correlated with overall survival in anaplastic oligodendroglioma, IDH1-mutant and 1p/19q-codeleted: A multicenter study from the French POLA network. Brain Pathol..

[58] Koster M.I., Kim S., Mills A.A., DeMayo F.J., Roop D.R. (2004). p63 is the molecular switch for initiation of an epithelial stratification program. Genes Dev..

[59] DeYoung M.P., Johannessen C.M., Leong C.O., Faquin W., Rocco J.W., Ellisen L.W. (2006). Tumor-specific p73 up-regulation mediates p63 dependence in squamous cell carcinoma. Cancer Res..

[60] Ramsey M.R., He L., Forster N., Ory B., Ellisen L.W. (2011). Physical association of HDAC1 and HDAC2 with p63 mediates transcriptional repression and tumor maintenance in squamous cell carcinoma. Cancer Res..

[61] Kukal S., Guin D., Rawat C., Bora S., Mishra M.K., Sharma P., Paul P.R., Kanojia N., Grewal G.K., Kukreti S. (2021). Multidrug efflux transporter ABCG2: Expression and regulation. Cell Mol. Life Sci..

[62] Wei L.Y., Wu Z.X., Yang Y., Zhao M., Ma X.Y., Li J.S., Yang D.H., Chen Z.S., Fan Y.F. (2020). Overexpression of ABCG2 confers resistance to pevonedistat, an NAE inhibitor. Exp. Cell Res..

[63] Kathawala R.J., Espitia C.M., Jones T.M., Islam S., Gupta P., Zhang Y.K., Chen Z.S., Carew J.S., Nawrocki S.T. (2020). ABCG2 Overexpression Contributes to Pevonedistat Resistance. Cancers.

[64] Hedberg M.L., Goh G., Chiosea S.I., Bauman J.E., Freilino M.L., Zeng Y., Wang L., Diergaarde B.B., Gooding W.E., Lui V.W. (2016). Genetic landscape of metastatic and recurrent head and neck squamous cell carcinoma. J. Clin. Investig..

[65] López-Arribillaga E., Rodilla V., Pellegrinet L., Guiu J., Iglesias M., Roman A.C., Gutarra S., González S., Muñoz-Cánoves P., Fernández-Salguero P. (2015). Bmi1 regulates murine intestinal stem cell proliferation and self-renewal downstream of Notch. Development.

[66] Jian X., He H., Zhu J., Zhang Q., Zheng Z., Liang X., Chen L., Yang M., Peng K., Zhang Z. (2020). Hsa_circ_001680 affects the proliferation and migration of CRC and mediates its chemoresistance by regulating BMI1 through miR-340. Mol. Cancer.

